# How learning style affects evidence-based medicine: a survey study

**DOI:** 10.1186/1472-6920-11-81

**Published:** 2011-10-08

**Authors:** Sandra E Zwolsman, Nynke van Dijk, Anita AH Verhoeven, Wouter de Ruijter, Margreet Wieringa-de Waard

**Affiliations:** 1 Department of General Practice/Family Medicine, Academic Medical Center-University of Amsterdam, Meibergdreef 15, 1105 AZ Amsterdam, the Netherlands; 2 Department of General Practice/Family Medicine, University Medical Center Groningen, University of Groningen, Hanzeplein 1, PO-Box 30.001, 9700 RB Groningen, the Netherlands; 3 Department of General Practice/Family Medicine, Leiden University Medical Center, Leiden University, Hippocratespad 21, 2333 ZD, Building 3, Leiden, the Netherlands

## Abstract

**Background:**

Learning styles determine how people manage new information. Evidence-based medicine (EBM) involves the management of information in clinical practice. As a consequence, the way in which a person uses EBM can be related to his or her learning style. In order to tailor EBM education to the individual learner, this study aims to determine whether there is a relationship between an individual's learning style and EBM competence (knowledge/skills, attitude, behaviour).

**Methods:**

In 2008, we conducted a survey among 140 novice GP trainees in order to assess their EBM competence and learning styles (Accommodator, Diverger, Assimilator, Converger, or mixed learning style).

**Results:**

The trainees' EBM knowledge/skills (scale 0-15; mean 6.8; 95%CI 6.4-7.2) were adequate and their attitudes towards EBM (scale 0-100; mean 63; 95%CI 61.3-64.3) were positive. We found no relationship between their knowledge/skills or attitudes and their learning styles (p = 0.21; p = 0.19). Of the trainees, 40% used guidelines to answer clinical questions and 55% agreed that the use of guidelines is the most appropriate way of applying EBM in general practice. Trainees preferred using evidence from summaries to using evidence from single studies. There were no differences in medical decision-making or in EBM use (p = 0.59) for the various learning styles. However, we did find a link between having an Accommodating or Converging learning style and making greater use of intuition. Moreover, trainees with different learning styles expressed different ideas about the optimal use of EBM in primary care.

**Conclusions:**

We found that EBM knowledge/skills and EBM attitudes did not differ with respect to the learning styles of GP trainees. However, we did find differences relating to the use of intuition and the trainees' ideas regarding the use of evidence in decision-making.

## Background

Evidence-based medicine (EBM) is defined as "the conscientious, explicit and judicious use of current best evidence in making decisions about the care of individual patients, in combination with the physician's clinical expertise, the patient's condition and the preferences of the patient" [1-4]. For teaching EBM to trainees, as with all forms of teaching, it is necessary to know whether the trainees differ in terms of their learning styles, prior knowledge of EBM and other EBM characteristics (attitudes and self-reported behaviour) [5]. Learning styles determine the management of new information [6]. EBM involves the management of information in clinical practice. As a consequence, the use of EBM could be related to a person's learning style. However, it is still unknown whether -based on this common link to the management of information- there is a relationship between learning styles and EBM. A potential relationship between learning styles and EBM would explain how trainees use information and combine this with their own preferences and with those of their patients in clinical decision-making.

### Theoretical framework

**Figure 1:**
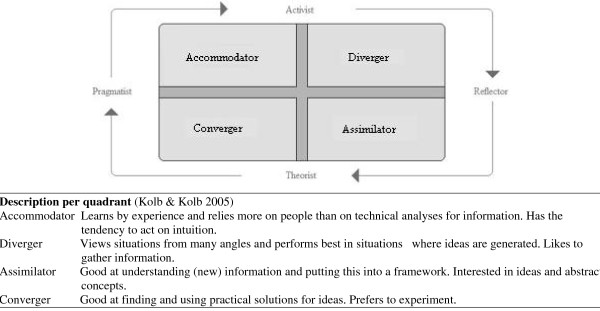
**Learning Style Quadrants**. Freely adapted from Kolb & Kolb 2005 and Honey & Mumford 1992

Kolb's experiential learning theory explains that learning style and problem-solving are closely related (Figure 1). Different factors (hereditary, experiential and environmental) contribute to a person's way of making decisions; that is, his or her preferred learning style. There are four learning styles, and most people prefer one style in particular, although some show mixed preferences. Learning style is measured on a bipolar perpendicular scale. Kolb & Kolb arranged the learning styles on two axes. The surface between the axes represents the four learning style quadrants: those of Accommodator, Diverger, Assimilator, and Converger. The largest surface (the quadrant of outcome) represents an individual's preferred learning style [6].

### Aim of the study

Education in EBM has been adopted by all of the Dutch General Practice Training Institutes. If a relationship between learning style and EBM were to be found, current educational approaches could be adapted to encourage trainees to develop their own particular way of making decisions (their preferred learning style) and to teach them how to improve their evidence-based decision-making in light of their own learning styles. The aim of this study is therefore to assess whether there is a relationship between learning style and predetermined EBM characteristics (knowledge/skills, attitude and self-reported behaviour).

## Methods

We conducted a cross-sectional survey study of 140 GP trainees who had just embarked on their Specialty Training. We collected data between September 2008 and May 2009.

### Setting

In the Netherlands, GP Specialty Training is a competency-based, three-year course consisting of clinical practice training and educational tutorials, organised in accordance with CanMEDS competencies at training institutes [7]. EBM plays an important role in this course, and trainees are given training in the knowledge/skills required for translating evidence into clinical practice [8,9].

### Subjects

In this study, we included first-year GP trainees from three GP training institutes (Leiden University, University of Groningen and AMC-University of Amsterdam) who were in the first month of their Specialty Training, prior to embarking on formal EBM education. The participating training institutes were selected from eight available institutes to ensure adequate representation in terms of geography and education.

### Study design

We simultaneously administered four questionnaires on EBM competence (personal characteristics, knowledge/skills, attitude, and behaviour) within each training institute under exam conditions (individually and under supervision). We printed the definition of EBM (as given in the introduction) at the top of each section of the questionnaire. The trainees filled in the learning-style questionnaire at home. We notified the trainees that we would use the questionnaires for research purposes, and all of the trainees gave written informed consent. The trainees were free to refrain from participation. The study was approved by the heads of the training institutes and was performed in accordance with the Declaration of Helsinki.

### Questionnaires

The trainees completed five questionnaires:

▪ *Personal characteristics: *besides sex, age and year of graduation, we asked the trainees about the previously attended EBM related courses, research experience, and how many years of experience they had in research and practice.

▪ *Learning style (*Figure 1*): *As in a recent study among GPs [10], Honey and Mumford's Learning Style Questionnaire [11] - translated into Dutch - was used to determine the trainees' learning styles. The questionnaire has been derived from Kolb's learning theory [6]. Kolb et al. arranged the learning styles along two axes: active-theoretical (vertical), and reflective-pragmatic (horizontal) [6]. The questionnaire consists of 80 true/false questions of which each learning style (active, theoretic, pragmatic and reflective) is represented by 20 questions [11]. Filling in the Learning Style Questionnaire leads to a score on each axis [11]. These scores on the four learning styles can be converted into Kolb's learning style quadrants that reflect the general preferred method of learning of a person and are related to the components (starting points) of Kolb's experiential learning cycle [6]. To calculate the learning style quadrants, we subdivided the outcomes of the questionnaire into the scores on the four learning style quadrants. We considered the quadrant with the highest outcome to be the trainee's preferred learning style, or, in the case of multiple quadrants, we designated the trainee to a mixed group (if scores in ≥2 quadrants were equal) [6,11].

▪ *Knowledge/skills: *to assess EBM knowledge/skills we used the two equivalent and interchangeable versions (version A and version B) of the Berlin questionnaire [12]. Each version consists of 15 multiple-choice questions and has good validity and reliability [12]. Each correct answer generates one point, with a minimum of 0 and a maximum of 15 points per questionnaire [12]. We translated and subsequently validated the Berlin questionnaire into Dutch, using forward-backward translation. In this study, we used the Dutch translation. The results of the validation of the Dutch Berlin questionnaire have been described elsewhere [13].

▪ *Attitude: *the attitude questionnaire, composed by McColl et al. [14], consists of 20 questions: seven with a visual analogue scale and 13 multiple-choice questions on an individual's general attitude towards EBM [14]. The questionnaire was translated into Dutch using forward-backward translation [15] and was adjusted for Dutch GP trainees. As the McColl questionnaire was developed in 1998, we adapted the questions on databases in line with current standards (Clinical Evidence, Trip, Sumsearch, Cochrane Systematic Reviews, PubMed Systematic Reviews via Clinical Queries). The overall attitude score from the visual analogue scales was valued between 0-100. We inversed scales with opposite outcome measures (questions 6 and 7) for statistical comparison, and determined the mean attitude. 

▪ *Self-reported behaviour: *the questionnaire on behaviour consists of five components, with a total of two open and 38 multiple-choice questions/statements about actual behaviour based on the "Conceptual Framework" of Straus et al. [16] and the five EBM steps as described by Dawes et al. [1]. We asked trainees about the factors that contribute to their clinical decision-making. For instance, we asked: "How often have you translated a clinical problem into an answerable question during the last two weeks?" For the statements, we used a 5-point Likert scale with: 1 = never, 2 = seldom, 3 = sometimes, 4 = often, and 5 = always.

### Statistical analysis

For statistical analyses, we used SPSS for Windows version 16.0. We described the characteristics of the participants and their overall scores on the questionnaires by means of proportions, means and 95% confidence intervals for continuous data that are normally distributed, and medians and interquartile range (quartiles) for data that are not normally distributed. We analysed all of the questionnaires as set out in the description of the original studies [6,11,12,14]. We compared differences in scores among subgroups for each learning style, using the χ^2 ^test for categorical data and ANOVA for continuous data that are normally distributed, and the Kruskal-Wallis test for data that are not normally distributed. We used a *post hoc *Bonferroni correction to deal with the bias of multiple testing.

We considered age [17], sex, previous EBM-related courses, research experience and years since graduation [18] to be potential confounders within learning style as related to EBM knowledge/skills, and EBM attitudes. As in previous studies, more experienced individuals (those who had graduated seven years or more previously) [17] and women [19] were shown to have higher levels of knowledge. We assumed possible confounding relations between the above-mentioned variables on the basis of research that indicates that there is a relationship between sex, research experience and time elapsed since graduation with knowledge [18] on the one hand, and a relationship between learning style and knowledge in postgraduate education [19] on the other. In the Netherlands, after having graduated from University, it is optional to either immediately start Specialty Training or -prior to Specialty Training- start obtaining practice experience or doing a PhD. As a result, there can be significant differences in practice experience and research experience between first-year trainees. Due to changes in EBM education, trainees who graduated during different periods may have received different kinds of education. We performed univariate and multivariate linear regression analyses to assess factors influencing knowledge/skills and attitudes towards EBM. Only knowledge/skills, and attitudes were taken into consideration, because these two variables have one numeric measure of outcome. This does not apply to the self-reported clinical behaviour variable. Only factors with a p-value of < 0.10 in the univariate analyses were entered into the multivariate model. Multivariate, we considered a p-value of < 0.05 to be statistically significant.

## Results

### Baseline characteristics

Of the 145 first-year GP trainees, 140 trainees were present during the questionnaire (a response rate of 97%): Amsterdam = 73/75, Leiden = 35/35 and Groningen = 32/35. Of these, 138/140 also filled in the learning-style questionnaire. The reasons for not responding were either absence during the administration of the questionnaire (5) or failure to hand in the learning-style questionnaire (2). The questionnaires were matched using the respondent's research number. Table 1 shows the characteristics of the participants. Of all GP trainees, 34% were Assimilators, followed by Convergers (20%), Accommodators (19%), and Divergers (14%). Trainees with two or more learning styles fell within the mixed group (14%). Of the trainees, 83% had followed an additional course in evidence-based searching (EBS) and 38% had taken a course in critical appraisal. Twelve per cent had completed a full EBM course. Course participation did not differ among groups with different preferred learning styles (Table 1).

**Table 1:** Baseline characteristics of 140 GP trainees and their learning styles

*Characteristics*		*All trainees*	*Accommodators*	*Divergers*	*Assimilators*	*Convergers*	*Mixed*
*Number of trainees*	n	140	26	19	47	28	20
*Women*	%	71%	69%	53%	77%	82%	60%
*Men*		29%	31%	47%	23%	18%	40%
*Age*	mean ± SD	29.3 ± 3.3	28.8 ± 2.4	30.6 ± 4.1	29.6 ± 3.8	28.8 ± 2.2	29.2 ± 3.6
*Years since graduation*	median IQR	2 (1-3)	2 (1-3)	2 (2-3)	2 (1-3)	2 (1-3)	1 (0.25-2)
Research experience*	%	28%	23%	16%	28%	33%	35%
*Practice experience*	%	90%	92%	100%	92%	86%	80%
Course EBS*	%	83%	77%	63%	89%	89%	90%
*Course Critical* Appraisal*	%	38%	24%	37%	41%	43%	40%
Course EBM*	%	12%	4%	11%	20%	7%	10%

*Not included in Medical Science studies at Bachelor's or Master's level

### EBM knowledge/skills Table 2

During the administration of the questionnaire, 52% filled in version A and 48% filled in version B of the Berlin questionnaire. The mean score of GP trainees was 6.8 (95% CI 6.4-7.2). The results for knowledge/skills were similar for the various learning styles.

**Table 2:** BM knowledge/skills and attitude in GP trainees

	*EBM knowledge/skills* *mean (95%CI)*	*EBM attitude* *mean (95%CI)*
*All trainees*	6.8 (6.4-7.2)	63 (61.3-64.3)
*Accommodator*	6.4 (5.6-7.2)	62 (58.3-65.5)
*Diverger*	7.7 (6.7-8.8)	61 (56.5-66.2)
*Assimilator*	6.4 (5.7-7.1)	62 (59.4-64.7)
*Converger*	7.1 (6.1-8.1)	63 (59.7-65.9)
*Mixed*	7.2 (5.9-8.4)	67 (63.1-71.5)

### EBM attitude Table 2

The overall attitude score, measured using the McColl questionnaire, points in a positive direction: the mean is 63 (95% CI 61.3-64.3) (0 = very negative, 100 = very positive). No difference was found in the mean attitude for trainees with different learning styles (p = 0.19).

### EBM self-reported behaviour: the EBM steps

#### Step 1: Ask

Of the participants, 52% reported that they had not asked themselves answerable questions on clinical problems encountered in patient consultations in the two weeks prior to filling in the questionnaire. The remaining participants, however, had asked themselves such questions and had subsequently answered them, although a majority (67%) did this in fewer than half of the clinical problems they had encountered. The various learning styles show no difference in self-reported behaviour (p = 0.45) (data not shown).

#### Step 2: Access

Most trainees (99%) have Internet access at home or at work, and only a small proportion of trainees do not have access to digital databases (10% at home and 7% at work). Table 3 shows where trainees seek information. When looking at use of information in clinical decision-making, Cochrane Systematic Reviews and/or PubMed Systematic Reviews were used by 52% of trainees, while synopses (Clinical Evidence, Trip Database and/or SUMsearch) were used by 3%. Of the trainees, 73.5% often asked their GP trainers for advice. There is no relationship between searching behaviour among trainees and having a particular preferred learning style.

**Table 3:** Information-seeking behaviour of 140 Dutch GP trainees

	*Clinical Guidelines for GPs*	*Systematic Reviews*	*Medline/PubMed*	*Ask GP trainer *
*Never*	-	40.9%	47.7%	0.7%
*Seldom*	-	26.8%	29.2%	-
*Sometimes*	2.2%	27.6%	16.9%	14.7%
*Often*	51.4%	4.7%	6.2%	73.5%
*Always*	46.4%	-	-	11.0%

As a response to the behaviour questionnaire, trainees currently prefer to use guidelines (40%) but indicate that in future, they would prefer to use reviews plus guidelines (37%), or would even prefer to learn how to fully apply EBM (38%). In practising EBM, all learning styles show comparable self-reported behaviour (p = 0.59). For general practice in particular, 55% believe that the exclusive use of guidelines is the preferred way to apply EBM. Assumptions about the way in which EBM should be used in general practice differ according to learning style (p = 0.04): Divergers and Convergers feel that using guidelines *plus *systematic reviews is best for general practice, while Accommodators and Assimilators think that using guidelines *alone *is sufficient.

Trainees do not necessarily record and file the answers they find when searching the literature: 14% regularly file questions and answers, 45% hardly ever do so and 41% never file questions and answers. If questions and answers are filed, trainees mainly do this by writing down annotations, and (sometimes) by using digital databases, saved files or printed results. No significant differences among the learning styles were found (p = 0.07).

#### Step 3: Appraise

When reading articles (Table 4), trainees focus on the abstract (median 4; interquartile range ((IQR) 1-4) on a 5-point Likert scale), but hardly ever read the whole article (score 2; IQR 1-2). If the entire article is read, the methods section or validity of the study is seldom appraised (score 1; IQR 1-3 and 1; IQR 1-2.75, respectively). Relevance is sometimes evaluated (score 3; IQR 1-4) and evidence from articles is occasionally applied to practice (score 2; IQR 1-4). Trainees with an Assimilating learning style score relatively high (though not significantly so) in all appraisal sections.

**Table 4:** Appraising articles per learning style quadrant

	*All trainees*	*Accommodators*	*Divergers*	*Assimilators*	*Convergers*	*Mixed*	*p-value*
*Methodology*	1 (1-3)	1 (1-3)	1 (1-2)	1 (1-3)	1 (1-2.25)	2 (1-3)	0.46
*Validity*	1 (1-2.75)	1 (1-2)	1 (1-2)	1 (1-3)	1 (1-2)	2 (1-3)	0.36
*Applicability*	2 (1-4)	1 (1-3)	2 (1-3)	3 (1-4)	2 (1-3.25)	2 (1-4)	0.59
*Relevance*	3 (1-4)	3 (1-4)	2 (1-3)	3 (1-4)	2 (1-3.25)	2.5 (1-4)	0.91

#### Step 4: Apply

When making clinical decisions (Table 5), the evidence retrieved (score 4; IQR 3-4), the trainees' clinical preferences (score 4; IQR 3-4), the trainers' preferences (score 4; IQR 3-4), and a patient's condition and prognosis (score 4; IQR 4-4 and 4; IQR 3-4) are taken into account. Patients' preferences (score 3; IQR 3-4) are considered less often. Intuition frequently plays a role in decision-making (score 4; IQR 3-4). When outcomes are split according to learning styles, the use of intuition differs significantly (p = 0.02): Accommodators and Convergers use their intuition in clinical decision-making more often.

**Table 5:** Variables influencing the decision-making process in GP trainees per learning style

	*All trainees*	*Accommodators*	*Divergers*	*Assimilators*	*Convergers*	*Mixed*	*p-value*
*Evidence *	4 (3-4)	4 (3.25-4)	4 (3-4)	4 (3-4)	4 (4-4)	4 (4-4)	0.17
*Trainees'* *intuition*	4 (3-4)	4 (3-4)	3 (3-4)	3 (3-4)	4 (4-4)	3 (3-4)	0.02
*Trainees' preferences*	4 (3-4)	4 (4-4)	3.5 (3-4)	4 (3-4)	4 (3-4)	3 (3-4)	0.49
*Trainers' preferences*	4 (3-4)	4 (3.25-4)	4 (3.75-4)	4 (3-4)	4 (4-4)	4 (3-4)	0.72
*Patients' preferences*	3 (3-4)	3 (3-4)	3 (3-3.25)	3 (3-4)	3 (3-4)	3 (2.25-4)	0.50
*Patients' condition*	4 (4-4)	4 (4-4)	4 (3-4)	4 (4-4)	4 (4-4)	4 (3.25-4)	0.79
*Patients'* *prognosis*	4 (3-4)	4 (3-4)	4 (3-4)	4 (3-4)	4 (3-4)	4 (3-4)	0.87

The evaluation of EBM performance was not assessed in this study.

### Multivariate analyses

None of the possible confounders (age, sex, previous EBM-related courses, research experience and number of years since graduation) were significantly related to EBM knowledge/skills and attitudes towards EBM.

## Discussion

When teaching EBM, it is necessary to have a clear idea of the characteristics of EBM learners [20]. We hypothesised that the manner in which individuals with their own preferred learning styles manage information could be related to these individuals' retrieval and use of new evidence (i.e. evidence-based practice). No significant differences in EBM knowledge/skills and EBM attitudes among novice trainees with their own preferred learning styles were found, however.

This does not imply that the learning style of trainees should not be taken into account when teaching the principles of EBM: we did find significant differences among the learning styles regarding trainees' ideas about the use of EBM in general practice, and how they use their intuition in their decision-making. Adapting teaching styles to learning styles may lead to improved motivation and learning on the part of trainees [21]. On the other hand, some studies suggest that adapting teaching styles to learning styles might not affect learning at all [22]. Considerable attention should thus be paid to the outcomes when adapting teaching styles to learning styles of trainees.

A particular point of interest is that the intuition of the trainees has a major influence on their decision-making. The fact that they reported frequent reliance on intuition when making clinical decisions is surprising, as one would not expect novice physicians to rely solely on intuition [23]. Experts who rely on intuition often apply internalised or integrated knowledge/skills [24], but it is unclear how trainees would do so. It would therefore be interesting to investigate what trainees understand by 'use of intuition' and to find out whether the decisions based on intuition are evidence-based. In education, particular attention should be paid to trainees with Accommodating and Converging learning styles, as they are more prone to using their intuition and thereby do not automatically work in an evidence-based way.

The trainees also had contrasting ideas about what constitutes the best evidence-based way of working. However, this relationship is more difficult to explain: Accommodators and Assimilators -opposites on the learning style axes- would rather use guidelines, whilst Convergers and Divergers would rather use guidelines plus systematic reviews. This outcome suggests that trainees with varying learning styles have different opinions about the evidence that they would want to use in their practice. Moreover, although extensive, the Dutch practice guidelines for General Practitioners are not always up-to-date and do not always answer every clinical question [9]. It would thus be interesting to know if and where Accommodators and Assimilators look for information that they cannot derive from the standards.

Although the above-mentioned differences among the learning styles are significant, one non-significant finding does require our attention: with respect to the asking and answering of questions that arise during clinical consultations, 67% of the trainees experienced this in fewer than 50% of the clinical problems they encountered. In other words: during practice consultations, trainees face uncertainties and gaps in their knowledge that are not filled by the relevant (scientific) information. The process of asking and answering questions was also studied by Ely et al., whose results confirm the finding in our study that GPs leave most questions unanswered [25].

There are some methodological limitations to the execution of this study. We used the outcomes of Honey and Mumford's Learning Style Questionnaire to calculate Kolb's Learning Style Quadrants [6,11]. In measuring learning styles, calculating the four basic learning styles (active, theoretic, pragmatic and reflective) using Honey and Mumford's Learning Style Questionnaire appears to be more reliable than using Kolb's inventory [21]. When using only the scores on the axes as outcomes, as suggested by Honey and Mumford, people obtain a score on each axis and have dominant scores in one or two of the basic learning styles [11,21]. However, in doing so, the integration between the dimensions of the axes is lost, and with them the relation with Kolb's experiential theory of learning, which is more informative for education. To overcome this problem, we calculated the quadrants Kolb originally suggested [6,10], indicating a generally preferred way(s) of learning for a person, relating to the starting point in Kolb's experiential learning cycle [26]. This information is more useful when coaching persons to apply effective methods of learning in specific situations [26]. That is why we -along with multiple studies on learning styles- calculate learning style quadrants to express the preferred learning style(s) of a person, instead of using the separate scores on both axes [10,11,19].

The lack of a relationship between a trainee's learning style and EBM learning might also be related to the validity of the learning-styles questionnaire and possible changes and flexibility in learning styles. Some doubt the validity and reliability of the division into different learning styles [26,27]. Other studies suggest that learning styles are highly dependent on the learning environment and the nature of the learning required [26,28,29]. The learning styles that the trainees present during formal training may therefore differ from the learning styles that they apply during clinical practice.

In addition, the assessment of EBM competence can be discussed. We assessed EBM behaviour using a self-reported measure, which may have influenced the outcomes. Currently, no valid measure, however, is available for measuring the actual practice of EBM [30]. Because the trainees filled in the questionnaires anonymously, we created optimal conditions to give reliable answers to the questions. The (lack of a) relationship between learning style and EBM behaviour should, however, be interpreted with caution until studies based on the observation of actual clinical behaviour have been performed. Our decision to use the Berlin questionnaire was based on the fact that this is the only available valid multiple-choice test on EBM knowledge/skills that measures the ability of applying knowledge in written clinical scenarios [30].

Overall, although the levels of knowledge/skills and attitudes of all GP trainees are comparable, these have different perceptions and ways of dealing with uncertainties in practice. Since evidence plays a critical role in GPs' clinical decision-making, and since the GP trainees' use of evidence depends on how they approach uncertainties, the differences found in this study need to be examined more closely. The differences found in this study regarding the use of intuition among the trainees could well form a critical barrier to the implementation of EBM. Therefore, since formal education is successful when it comes to providing trainees with EBM knowledge/skills, and since the manner in which GP trainees make decisions is ultimately affected by their learning styles, attention should be paid to transferring these knowledge/skills to clinical practice. In EBM education, teachers should therefore focus more on providing trainees with the skills needed to combine evidence, their personal preferences as doctors, and the preferences of the patient [31]. As a result, a shift from formal training in the five steps [1] towards EBM decision-making in practice should become a priority in the development of EBM courses.

## Conclusions

Learning styles are not related to the EBM competence of novice GP trainees. However, significant differences among the trainees were found regarding their use of intuition in decision-making, the way in which they sought answers to their questions, and their perception of what constituted the best way of applying EBM in general practice. Research in clinical practice needs to be undertaken in order to understand how trainees handle information in practice, and what kind of practical education could improve the actual EBM behaviour of GP trainees.

## Competing interests

The authors declare that they have no competing interests.

## Authors' contributions (in conformity with ICMJE)

SEZ was the primary investigator of this study and 1) designed the study, acquired the data and analysed and interpreted the data; 2) drafted the article; and 3) approved the final version to be published.

NvD was the research supervisor of the study and 1) designed the study, assisted in data analysis and interpretation of the data; 2) revised the article critically for important intellectual content; and 3) approved the final version to be published.

AAHV 1) made substantial contributions to the acquisition of data; 2) revised the article critically for important intellectual content; and 3) approved the final version to be published.

WdR 1) made substantial contributions to the acquisition of data; 2) revised the article critically for important intellectual content; and 3) approved the final version to be published.

MWdW was the clinical supervisor of the study and 1) designed the study and assisted in the acquisition and interpretation of data; 2) revised the article critically for important intellectual content; and 3) approved the final version to be published.

## Pre-publication history

The pre-publication history for this paper can be accessed here:

http://www.biomedcentral.com/1472-6920/11/81/prepub
